# Characterization and Antitumor Activity of a Polysaccharide from *Sarcodia ceylonensis*

**DOI:** 10.3390/molecules190810863

**Published:** 2014-07-25

**Authors:** Yijun Fan, Mengchuan Lin, Aoshuang Luo, Ze Chun, Aoxue Luo

**Affiliations:** 1 Department of Landscape Plants, Sichuan Agriculture University, Chengdu 611130, China; 2 Chengdu Institute of Biology, Chinese Academy of Sciences, Chengdu 610041, China

**Keywords:** *Sarcodia ceylonensis*, polysaccharide, antitumor, purification

## Abstract

A water-soluble polysaccharide from *Sarcodia ceylonensis* was obtained by using the method of water-extraction and ethanol-precipitation. The polysaccharide was further purified by chromatography on AB-8 and ADS-7 columns, yielding a pure polysaccharide termed SCP-60. The molecular weight (Mw) of SCP-60 was calculated to be 50.0 kDa, based on the calibration curve obtained with a series of Dextran T standards. The results of FT-IR indicated that the polysaccharide contains the α-configuration of sugar units. GC-MS analysis revealed that SCP-60 was mainly composed of galactose and glucose. NMR spectroscopy revealed SCP-60 had the backbone consisting of →6)-α-Manp-(1→, α-d-Glcp-(1→, →6)-α-d-Glcp-(1→ and →6)-α-Galp-(1→. In order to evaluate the antitumor activity *in vivo* of the polysaccharide, a sarcoma 180 model was used. The results showed SCP-60 had strong antitumor ability, meanwhile, SCP-60 at a high dose (100 mg/kg) could significantly increase the thymic and splenic indices of S180 mice, and strongly promote the secretion of IL-2, TNF-α and IFN-γ, increase the SOD activities and reduce the concentrations of MDA in blood. Therefore the polysaccharide SCP-60 should be explored as a novel potential antitumor drug.

## 1. Introduction

Polysaccharides are not only energy resources, but play key biological roles in many life processes as well. The structures and mechanisms of pharmaceutical effects of bioactive polysaccharides on some diseases have been extensively studied. Most polysaccharides are relatively nontoxic and do not cause significant side effects, which is a major problem associated with synthetic compounds. Thus, polysaccharides are ideal candidates for therapeutics with immunomodulatory, antitumor and wound-healing action [1,2]. Seaweeds are the most abundant source of polysaccharides, such as alginates, agar and agarose as well as carrageenans. Some polysaccharides from marine algae exhibit beneficial biological properties, such as a polysaccharide from *Gracilaria birdiae*, which can prevent naproxen-induced gastrointestinal damage in rats [3]. Sulfated polysaccharides isolated from *Sphaerococcus coronopifolius* (*Rhodophytha*, *Gigartinales*) and *Boergeseniella thuyoides* (*Rhodophyta*, *Ceramiales*) have strong antiviral activities [4]. Polysaccharides in marine sponges have anti-HIV activity [5]. In addition, some polysaccharides from marine algae such as *Sargassum mcclurei* [6], *Undaria pinnatifida* [7] and the brown alga [8] have significant anticancer properties.

Immune system dysfunction is responsible for various diseases, such as cancer and infectious diseases. Most reports confirmed that polysaccharides exerted their anti-tumor effect because of the activation of the immune system of the host animal [9,10]. As the important immune organs, the spleen indices and thymus indices reflect the immune function of the organism [11]. Recently polysaccharides have been used as a source of therapeutic agents for cancer due to their relatively low toxicity and antitumor activities through the immune response of the host organism [12]. *Sarcodia ceylonensis* is a red algae and commonly consumed as seafood, and used as as a medical resource for its hypolipidemic and immune enhancement effect [13]. There are abundant polysaccharides in *Sarcodia ceylonensis*, however, until now, the purification and biological activity of the polysaccharides from *Sarcodia ceylonensis* has not been reported. Therefore, the purpose of the present investigation was to elucidate the isolation and characterization of a water-soluble polysaccharide from *Sarcodia ceylonensis*, as well as to evaluate its anticancer activity *in vivo*.

## 2. Results and Discussion

### 2.1. Polysaccharide Isolation and Purification

The crude polysaccharide SCP-60 was obtained by using the method of water-extraction and ethanol-precipitation. Because of the presence of some colored materials and residual protein, the crude polysaccharide was a yellow water-soluble powder. AB-8 and ADS-7 macroporous resins were used for further purification of the polysaccharides. After application of these two macroporous adsorption resins, the polysaccharide was a gray-colored powder appearance. The molecular weight (Mw) of the polysaccharide SCP-60 was calculated to be 50.0 KDa, based on the calibration curve obtained with a series of Dextran T standards. 

Monosaccharide composition of SCP-60 was analyzed by the trifluoroacetic acid (TFA) hydrolysis and GC-MS analysis. The results indicate that galactose, glucose, xylose and mannose were the major monosaccharide constituents. The total content of monosaccharide compositions in SCP-60 was described as follows: galactose: glucose: xylose: mannose = 50.78%: 26.15%: 9.55%: 13.52%.

### 2.2. Infrared Spectrum of SCP-60

**Figure 1:**
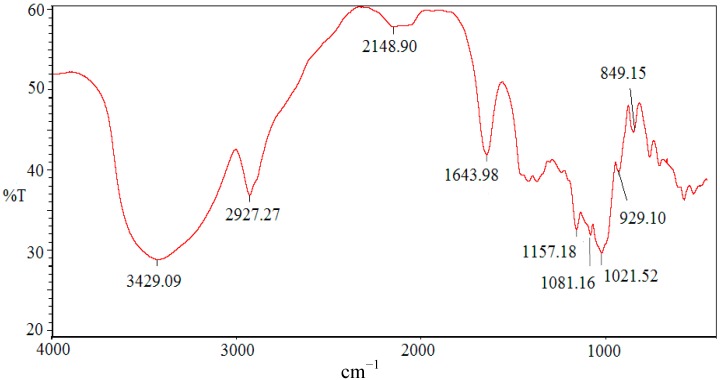
IR spectrum of the polysaccharide SCP-60.

The FT-IR spectrum of SCP-60 is presented in Figure 1. In the spectrum, the band in the 3429.09 region corresponds to the hydroxyl stretching vibration, while the band at 2927.27 corresponds to a weak C–H stretching vibration [14], which indicates that SCP-60 is a polysaccharide. The band at 1643.98 is due to the bound water [15]. The polysaccharide had a specific band in the 1200–1000 cm^−1^ region, which is dominated by ring vibrations overlapped with stretching vibrations of (C-OH) side groups and the (C-O-C) glycosidic band vibrations [16]. The positive specific rotation and the characteristic absorption at 849.15 cm^‒1^ in SCP-60 indicated the α-configuration of the sugar units [17]. 

### 2.3. NMR Identification of SCP-60

**Figure 2:**
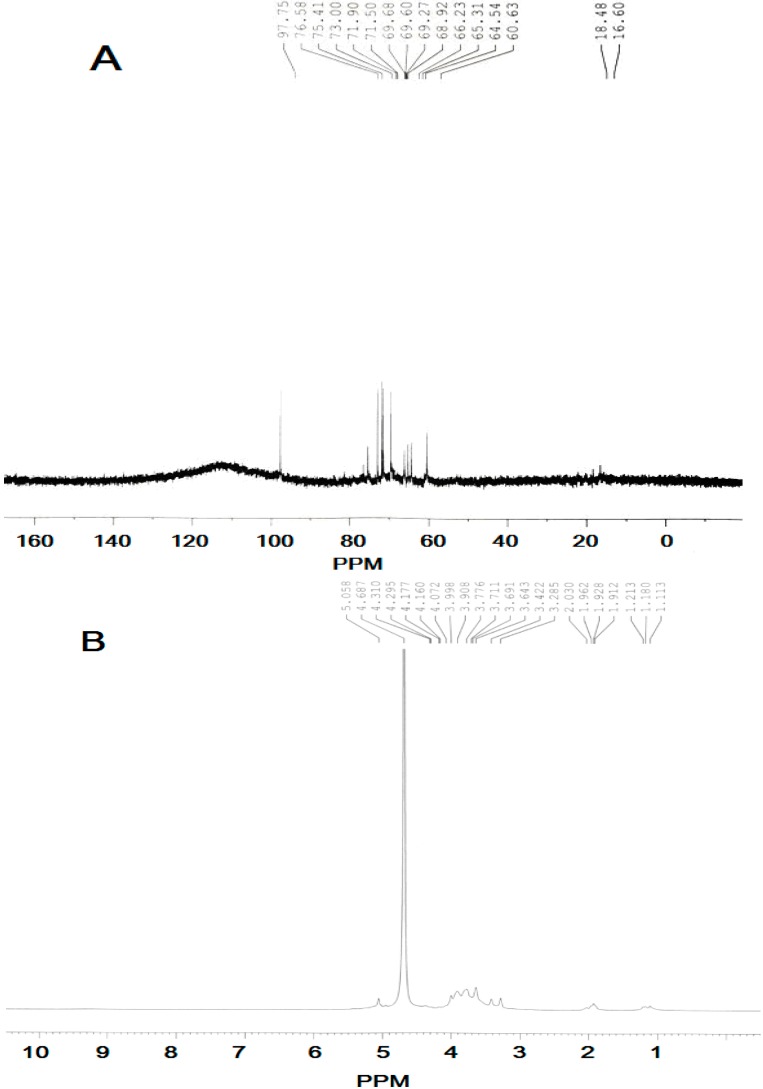
NMR analysis of SCP-60. (**A**) ^13^C-NMR of SCP-60, and (**B**) ^1^H-NMR analysis of SCP-60.

The ^1^H-NMR and ^13^C-NMR results for SCP-60 were assigned by comparison with the previously reported NMR data and detailed assignments of all signals are shown in Table 1. Based on previous discussions in the literature, chemical shifts between δ 98 and 103 ppm are a typical feature of the C-1 in α-glycosidic linkages, whereas in the case of β-glycosidic linkages signals would be expected between δ 103 and 106 ppm [18,19]*.* Thus, the signals were δ 97.75 indicating α anomeric configuration for all monosaccharide residues of SCP-60 (Figure 2). These results indicated that SCP-60 was composed of a repeating unit with the possible main structure as follows:

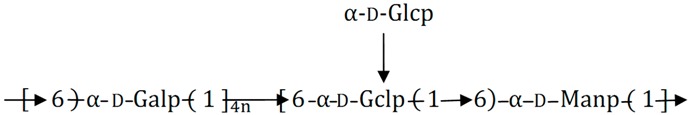


**Table 1:** ^1^H-NMR and ^13^C-NMR chemical shifts for SCP-60 in D_2_O at 27°C.

Sugar Residues	C-1	C-2	C-3	C-4	C-5	C-6
	H-1	H-2	H-3	H-4	H-5	H-6
α-d-Glcp-(1→	97.75	71.50	75.41	69.67	73.00	60.63
	5.058	3.711	3.776	3.643	3.908	3.908
→6)-α- Manp-(1→	97.75	75.41	71.50	68.92	71.90	66.23
	5.058	4.072	3.908	3.776	3.711	3.998
→6)-α-d-Glcp-(1→	97.75	73.00	75.41	69.68	71.90	68.92
	5.058	3.643	3.776	3.422	3.908	3.998
→6)-α-Galp-(1→	97.75	68.92	69.68	68.92	69.60	66.23
	5.058	3.711	3.776	3.908	3.998	3.691

### 2.4. Tumor Inhibition Effects

Although tumor inhibition rate was not the only standard of anti-tumor activity, tumor mass reduction often indicates a strong anti-tumor effect, so tumor inhibition rate is usually used as an index for screening anti-tumor drugs. Cyclophosphamide (CTX) is generally used for treatment of various types of cancers. It is a clinically approved anticancer agent that works by slowing or stopping cell growth. Table 2 shows that increasing concentrations of SCP-60 resulted in increased rates of tumor inhibition. Maximum inhibition (65.62 ± 3.29%) was observed at 100 mg/mL of SCP-60, which was comparable to the levels of tumor inhibition exhibited by the positive control, CTX (20 mg/mL).

**Table 2:** Tumor inhibition effect and the analysis of immune index.

Group	Dose (mg/kg)	Inhibition Rate of Tumor (%)	Thymus Index(mg/g)	Spleen Index(mg/g)
MC	‒	‒	3.01 ± 0.66	12.99 ± 0.97
CTX	20	65.62 ± 3.29	1.13 ± 0.05	8.72 ± 0.55
Polysaccharide	100	69.01 ± 5.01	5.69 ± 0.37 ^bc^	16.79 ± 1.09 ^bc^
	50	57.95 ± 3.75	4.85 ± 0.21 ^ac^	15.37 ± 1.16 ^bc^
	25	36.78 ± 2.22	3.78 ± 0.09 ^c^	13.06 ± 0.86 ^c^

Each value represents mean ± SD (*n* = 10); ^a^ *p* < 0.05 (compared with model control); ^b^ *p* < 0.01 (compared with model control); ^c^ *p* < 0.01 (compared with CTX group).

### 2.5. Analysis of Immune Index

The immune system has the capacity either to prevent tumor development and restrain established tumors, or to promote tumorigenesis, tumor progression and metastasis [20]. To evaluate the effect of the polysaccharide SCP-60 on the immune system, the effects on splenic and thymic indices of different samples were evaluated in S180 tumor-bearing mice. As seen from the Table 2, different doses of polysaccharide exhibited dose-dependent effects on splenic and thymic indices with the lowest dose (25 mg/mL) having no significant effect and the highest dose (100 mg/mL) causing the greatest increases, significantly higher than the model control group and the CTX group. At 100 mg/kg SCP-60, the thymic and splenic indices were 5.69 ± 0.37 mg/g and 16.79 ± 1.09 mg/g, respectively, Such increases may indicate a positive effect on the immune system and may at least partially explain the antitumor effect of SCP-60. In contrast, the thymic and splenic indices were decreased in mice treated with CTX, suggesting a negative affect on the immune system. 

### 2.6. Effect of SCP-60 on Body Weight in Mice

**Figure 3:**
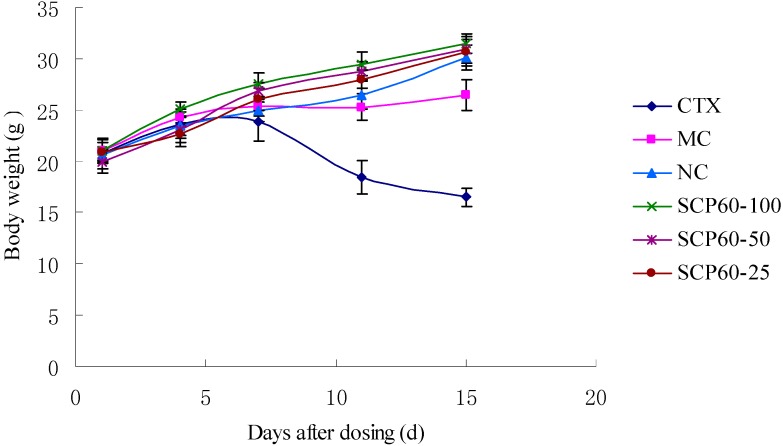
Effects of different doses of SCP-60 and CTX on body weight in S180 mice. NC: normal control group. MC: model control.

Before embarking on the experiments, the body weights of all the groups were 20.0 ± 2.0 g. There were no significant difference in body weight each groups (*p* > 0.05; Figure 3). After drug injection, there were significant (*p <* 0.05) decreases in body weight detected in the model control and CTX group. Especially in the CTX group, many mice exhibited ruffled fur, lethargy and loss of appetite, which suggested some toxic effect of CTX on S180 mice. On the other hand, the body weights in the SCP-60 groups were significantly (*p <* 0.05) and dose-dependently increased, as compared to those of the normal control group from 4 days after administration. The results showed that there was no significant toxic effect of the polysaccharide SCP-60 to S180 mice.

### 2.7. Determination of IL-2, TNF-α, and IFN-γ using ELISA Kits

Serum from mice from each groups were collected 4 h after administration of the drugs. The interleukin-2 (IL-2), tumor necrosis factor-alpha (TNF-α) and interferon-gamma (IFN-γ) concentrations were measured with an enzyme-linked immunosorbent assay according to the instructions of the manufacturer. IFN-γ is an important immunoregulatory molecule. Both TNF-α and IFN-γ are known to have significant antitumor activities through a variety of mechanisms that are often immunological. 

**Figure 4:**
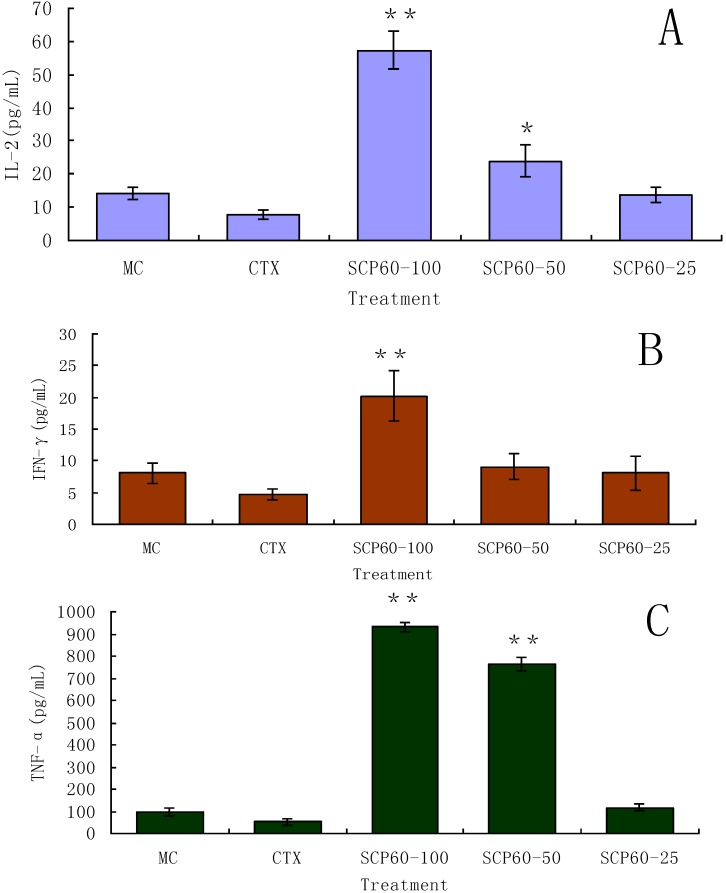
Effects of different doses of SCP-60 and CTX on the secretion of three cytokines. (**A**) for IL-2, (**B**) for IFN-γ and (**C**) for TNF-α. Values are means ± S.D., (*n* = 10); ** *p* < 0.01, * *p* < 0.05 *vs.* model control.

From the results of Figure 4, the concentrations of IL-2, TNF-α and IFN-γ in the group treated with CTX were significant low, which indicated CTX could not promote the secretion of the three cytokines. However, SCP-60 at 100 mg/kg, strongly promoted the secretion of IL-2, TNF-α and IFN-γ (*p* < 0.01) compared with the model control. At 50 mg/kg, the effects of polysaccharide on promoting the secretion of TNF-α (*p* < 0.01) and IL-2 (*p* < 0.05) were significant. At 25 mg/kg SCP-60, none of the cytokines were significantly increased to levels above the untreated control.

### 2.8. Analysis of SOD and MDA in S180 Mice

Oxidative stress induced by free radicals is known to cause serious illnesses including heart disease and cancer. Free radicals especially superoxide anion radicals are produced in cancer cells more than what seen in normal cells. Therefore, in recent years, there has been a mounting interest in the use of natural products with antioxidant effects in the management of cancer. Antioxidant enzymes are considered to be a primary defense that prevent biological macromolecules from damage. SOD is an important enzymatic antioxidant enzyme in antioxidant systems, which changes superoxide anion radicals to hydrogen peroxide which in turn, decomposes to water and oxygen by glutathione peroxidase and catalase. Therefore, enhanced activity of SOD was considered to play an important role as a dietary radical scavenger for the prevention of oxidative damage to DNA chains [21]. As shown in Table 3, the SOD activity in the model control was very poor, which indicated that the ability of antioxidant system in the mice was decreased upon tumor inoculation.

**Table 3:** Analysis of SOD and MDA in S180 mice.

Group	Dose (mg/kg)	SOD (U/mL)	MDA (nmol/mL)
MC	‒	34.69 ± 3.17	30.51 ± 3.66
CTX	20	38.34 ± 2.77	41.69 ± 4.29
Polysaccharide	100	63.63 ± 4.82 ^bc^	7.62 ± 1.03 ^bc^
	50	52.15 ± 3.62 ^bc^	10.12 ± 2.77 ^bc^
	25	41.71 ± 3.86 ^a^	26.57 ± 2.82 ^c^

Each value represents mean ± SD (*n* = 10); ^a^ *p* < 0.05 (compared with model control); ^b^ *p* < 0.01 (compared with model control); ^c^ *p* < 0.01 (compared with CTX group).

On the other hand, the SOD activity in the polysaccharide-treated mice was excellent, especially at the high doses, at 100 mg/kg, it was far higher than CTX-treated and model control groups. Therefore the results suggested that the polysaccharide could increase the SOD activities in S180 mice. 

Malondialdehyde (MDA) is formed during oxidative degeneration as a product of free oxygen radicals [22], which is accepted as an indicator of lipid peroxidation [23]. MDA levels were reported to be higher in cancer tissues than in non-diseased organs [24]. It was reported that plant-derived extracts containing antioxidant properties showed cytotoxicity towards tumor cells [25]. Antitumor activity of these antioxidants is either through induction of apoptosis or by inhibition of neovascularization [26]. The free radical hypothesis supported by the fact that antioxidants can effectively inhibit tumor growth [27]. The MDA value was estimated according to the thiobarbituric acid method [28]. The samples added with TBA were heated in an acidic environment. The absorbance of the resulting solution was measured at 532 nm. The results in Table 3 show a significant pattern of a decreasing MDA concentration in all polysaccharide-treated groups. At 100 mg/kg, the concentration of MDA was the lowest than that of all other groups. This can be interpreted as a significant effect of SCP-60 on MDA scavenging in S180 mice.

## 3. Experimental Section

### 3.1. Materials and Chemicals

Dextrans of different molecular weights were purchased from Pharmacia Co. (Uppsala, Sweden). Cyclophosphamide (CTX) was purchased from Hengrui Medicine Co. (Jiangsu, China). The monosaccharide standards (glucose, mannose, rhamnose, galactose, xylose and arabinose) were purchased from the Chinese Institute for the Control of Pharmaceutical and Biological Products (Beijing, China). The tumor necrosis factor-alpha (TNF-α), interferon-gamma (IFN-γ) and interleukin-2 (IL-2) enzyme-linked immunosorbent assay (ELISA) kits were purchased from Shanghai Senxiong Biotech Co. (Shanghai, China). AB-8 and ADS-7 were purchased from the Chemical Plant of Nankai University (Tianjin, China). Trifluoroacetic acid (TFA), pyridine, methanol, and acetic acid, ethanol, acetic anhydride and all other chemicals and reagents were purchased from Chengdu Kelong Chemical (Chengdu, China).

### 3.2. Extraction of Crude Polysaccharide from Sarcodia ceylonensis

The *Sarcodia ceylonensis* were thoroughly washed with water, dried at 60 °C, and then powdered with a pulverizer. The powder was extracted in six times the volume (v/m) of 0.4% HCl for 8 h. The solution was separated from *Sarcodia ceylonensis* residues by successive filtration through gauze, and the residues were extracted with 0.4% HCl (six times the volume) three times. All extracts were combined, neutralized with NaOH, and filtered. Then all extracts were concentrated using a rotary evaporator at 55 °C. The extract was deproteinized four times using the Sevag reagent [29]. After removal of the Sevag reagent, the extract was precipitated by adding ethanol (two-thirds times the volume of aqueous extract), and the mixture was kept overnight at 4 °C. After filtration and centrifugation, the solution was successively precipitated by adding ethanol until the concentration of ethanol got to 60% to yield the crude polysaccharide (named SCP-60). After washed successively with ethyl acetate and acetone, the polysaccharide was dissolved in double-distilled water and subsequently dialyzed against deionized water for 72 h and lyophilized.

### 3.3. Purification of the Crude Polysaccharide

Purification of the crude polysaccharide SCP-60 was performed according to the method of Luo *et al.* [30]. Two macroporous adsorption resins, AB-8 and ADS-7, were used for purifying the crude polysaccharide. AB-8 was selected for the decoloration and ADS-7 was used to remove the residual protein. First, the crude polysaccharide was dissolved in double-distilled water. After membrane filtration (0.45 μm, Nucleopore, CT, USA), the filtrate was injected to columns (26 × 300 mm) of AB-8 and ADS-7, respectively. The columns were eluted with distilled water. Protein content was determined according to the method of Bradford *et al.* [31], with bovine serum albumin as a standard. 

### 3.4. Determination of the Molecular Weights of Purification Polysaccharide

The molecular weights of the polysaccharide SCP-60 was determined by the Gel Permeation Chromatography according to the method of Yamamoto *et al.* [32], in combination with a Waters 515 HPLC (Waters, Milford, MA, USA) equipped with a Ultrahydrogel Linear Column (300 × 7.8 mm). The column was eluted with 0.2 M phosphate buffer (pH 7.0) at a flow rate of 0.7 mL/min and detected by a Waters 2410 refractive index detector (RID). Dextran standards with different molecular weights (2500, 4600, 7100, 10,000, 21,400, 41,100, 84,400, 133,800, 200,000 Da) were used to plot the calibration curve.

### 3.5. Infrared Spectra Analysis

The structural characteristics of SCP-60 was determined on a Fourier transform IR spectrophotometer (Perkin-Elmer Corp., Waltham, MA, USA). The polysaccharide was ground with KBr powder and then pressed into pellets for IR measurements in the frequency range of 4000–500 cm^−1^ [33].

### 3.6. Analysis of Monosaccharide Compositions

SCP-60 (10.0 mg) was hydrolyzed with 2.0 M TFA at 110 °C for 4 h in a sealed glass tube. Then the hydrolysate was evaporated to dryness and dissolved in pyridine (0.5 mL), then hydroxylamine hydrochloride (10.0 mg) and myo-inositol (2.0 mg, as internal reference) were added to the solution, it was allowed to react at 90 °C for 30 min. The tube was cooled to room temperature, and then 0.5 mL of acetic anhydride was added and mixed thoroughly by vortexing. The tube was sealed and incubated in a water bath shaker set at 90 °C for 30 min. After cooled, approximately 1.0 μL of clear supernatant was loaded onto an Rtx-5SilMS column (30 m × 0.32 mm × 0.25 μm) of the GC-MS. Alditol acetates of authentic standards (glucose, mannose, rhamnose, galactose, xylose and arabinose) with myo-inositol as the internal standards were prepared and subjected to GC-MS analysis separately in the same way [34,35].

### 3.7. NMR Identification

Twenty milligrams of SCP-60 was dissolved in D_2_O (0.5 mL, 99.9%), freeze-dried, and redissolved in D_2_O (0.5 mL). The ^1^H-NMR and ^13^C-NMR spectra were measured in an NMR tube (5 mm diameter) at 27 °C with a Bruker Avance 600 spectrometer. The chemical shift was expressed in parts per million (ppm).

### 3.8. Antitumor Test in Vivo

#### 3.8.1. Animals and Treatment

Kunming mice (weight: 20.0 ± 2.0 g) between 6 and 8 weeks old were housed under normal laboratory conditions, *i.e.*, room temperature, 12/12 h light–dark cycle with free access to standard rodent chow and water. Sarcoma 180 cells were passed into mice abdominal cavity. Then, ascites was inoculated subcutaneously 0.2 mL (5.0 × 10^7^ cells/mL) into the sword arm of each experimental mouse. The mice were treated as following: positive control (CTX, 20 mg/kg body weight), model control group (normal saline) and the polysaccharide (100 mg/kg, 50 mg/kg and 25 mg/kg body weight). All the groups were administered daily by intraperitoneal injection. 

#### 3.8.2. Tumor Inhibition Effect

The tested samples (100 mg/kg, 50 mg/kg and 25 mg/kg body weight) and CTX (20 mg/kg body weight) were dissolved in saline, and then injected intraperitoneally (i.p.) once a day for 14 days, starting 24 h after tumor inoculation. The normal control and model control mice received an equal volume of saline (0.2 mL). 24 h after last tested sample administration. All animals were weighted and sacrificed. The inhibitory rate was calculated as [(A − B)/A] × 100% [36], where A and B were the average tumor weights of the model group and the tested group, respectively.

#### 3.8.3. Analysis of Immune Index

Splenic index was determined from the weight of the spleen of the mice surviving up to 14 days. Twenty-four hours after the last administration, these mice were euthanized by cervical dislocation, and the spleens were recovered and weighed. The results are expressed as the spleen index using the formula: weight of spleen (mg)/ body weight (g). The thymic index and the tumor weights of the mice were measured by using a similar method.

#### 3.8.4. Determination of IL-2, TNF-α, and IFN-γ by ELISA Method

These mice were euthanized after 14 d, and their blood samples were obtained from eye orbitae. Serum was collected 4 h after administration of the polysaccharide. The interleukin-2, tumor necrosis factor-alpha, and interferon-gamma concentration were measured with an enzyme-linked immunosorbent assay according to the indication of the manufacturer.

#### 3.8.5. Detection of SOD Activity and MDA Level in S180-Bearing Mice

Twenty-four hours after the last drug administration, blood samples were obtained from the eye pit of the mice and processed for serum. The superoxide dismutase (SOD) activity and the malondialdehyde (MDA) level were also measured. SOD activity (U/mL) was tested with the SOD assay kit.

### 3.9. Statistical Analysis

The data were presented as mean ± standard deviation. Significance of difference was evaluated with one way analysis of variance (ANOVA) by the SPSS 16.0 software package. The values were considered significant when *p* < 0.05.

## 4. Conclusions

According to the results described above, the water-extracted crude polysaccharide SCP-60 from *Sarcodia ceylonensis* was obtained by using the methods of water-extraction and ethanol-precipitation. In order to yield highly purified polysaccharide, two macroporous adsorption resins AB-8 and ADS-7 were used. SCP-60 exhibited strong anti-tumor activity *in vivo*, while SCP-60 at 100 mg/ kg also significantly increased the thymic and spleen indices of S180 mice, and strongly promoted the secretion of IL-2, TNF-α and IFN-γ. Because IL-2 can promote the long-term proliferation of T cells, TNF-α and IFN-γ can enhance immunoregulatory ability each other towards anti-tumor. Therefore, enhancement of the cellular arm of the immune response may be one possible mechanism by which SCP-60 may have inhibited tumor growth. In addition, SCP-60 demonstrated a strong capacity to reduce the concentrations of MDA in blood serum from mice, which suggests that preventing lipid peroxidation *in vivo* is another possible mechanism by which SCP-60 inhibits tumor growth. Further investigation on mechanism of SCP-60 antitumor activities *in vivo* will be carried out in our later work. And with the results above, SCP-60 could be explored as a potential antitumor drug.

## References

[1] Schepetkin I.A., Quinn M.T. (2006). Botanical polysaccharides: Macrophage immunomodulation and therapeutic potential. Int. Immunopharmacol..

[2] Fan Y.J., Luo A.X. (2011). Evaluation of anti-tumor activity of water-soluble polysaccharides from Dendrobium denneanum. Afr. J. Pharm. Pharm..

[3] Silva R.O., Santana A.P.M., Carvalho N.S., Bezerra T.S., Oliveira C.B., Damasceno S.R.B., Chaves L.S., Freitas A.L.P., Soares P.M.G., Souza M.H.L.P. (2012). A sulfated-polysaccharide fraction from seaweed gracilaria birdiae prevents naproxen-induced gastrointestinal damage in rats. Mar. Drugs.

[4] Bouhlal R., Haslin C., Chermann J.C., Colliec-Jouault S., Sinquin C., Simon G., Cerantola S., Riadi H., Bourgougnon N. (2011). Antiviral activities of sulfated polysaccharides isolated from *Sphaerococcus coronopifolius* (Rhodophytha, Gigartinales) and *Boergeseniella thuyoides* (Rhodophyta, Ceramiales). Mar. Drugs.

[5] Esteves A.I.S., Nicolai M., Humanes M., Goncalves J. (2011). Sulfated polysaccharides in marine sponges: Extraction methods and anti-hiv activity. Mar. Drugs.

[6] Thinh P.D., Menshova R.V., Ermakova S.P., Anastyuk S.D., Ly B.M., Zvyagintseva T.N. (2013). Structural characteristics and anticancer activity of fucoidan from the Brown Alga *Sargassum mcclurei*. Mar. Drugs.

[7] Maruyama H., Tamauchi H., Hashimoto M., Nakano T. (2003). Antitumor activity and immune response of mekabu fucoidan extracted from sporophyll of *Undaria pinnatifida*. In Vivo.

[8] Eldeen A.M.G., Ahmed E.F., Zeid M.A.A. (2009). *In vitro* cancer chemopreventive properties of polysaccharide extract from the brown alga, *Sagassum latifolium*. Food Chem. Toxicol..

[9] Chen X., Nie W., Yu G., Li Y., Hu Y., Lu J., Jin L. (2012). Antitumor and immunomodulatory activity of polysaccharides from *Sargassum fusiforme*. Food Chem. Toxicol..

[10] Han X.Q., Wu X.M., Chai X.Y., Chen D., Dai H., Dong H.L., Ma Z.Z., Gao X.M., Tu P.F. (2011). Isolation, characterization and immunological activity of a polysaccharide from the fruit bodies of an edible mushroom, *Sarcodon aspratus* (Berk.) S. Ito. Food Res. Int..

[11] Zhao T., Mao G.H., Mao R., Zou Y., Zheng D.H., Feng W.W., Ren M., Wang W., Zheng W., Song J. (2013). Antitumor and immunomodulatory activity of a water-soluble low molecular weight polysaccharide from *Schisandra chinensis* (Turcz.) Baill. Food Chem. Toxicol..

[12] Wu X., Mao G., Fan Q., Zhao T., Zhao J., Li F., Yang L. (2012). Isolation, purification, immunological and anti-tumor activities of polysaccharides from *Gymnema sylvestre*. Food Res. Int..

[13] Xia B.M. (1999). Chinese Journal of Marine Algae Volume II Rhodophyta.

[14] Luo D.H. (2008). Identification of structure and antioxidant activity of a fraction of polysaccharide purified from *Dioscorea nipponica Makino*. Carbohydr. Polym..

[15] Liu Y.H., Wang F.S. (2007). Structural characterization of an active polysaccharide from *Phellinus ribis*. Carbohydr. Polym..

[16] Zhao M.M., Yang N., Yang B. (2007). Structural characterization of water-soluble olysaccharides from *Opuntia monacanthap cladodes* in relation to their anti-glycated activities. Food Chem..

[17] Barker S.A., Bourne E.J., Stacey M., Whiffen D.H. (1954). Infrared spectra of carbohydrates. Part I. Some derivatives of d-glucopyranose. J. Chem. Soc..

[18] Yoon S., Kim M.K., Lee I.Y., Yun M., Nam Shin J.E. (2008). Production and structural features of a water-soluble polysaccharide from a mutant strain of *Agrobacterium* sp.. J. Ind. Eng. Chem..

[19] Kath F., Kulicke W.M. (1999). Mild enzymatic isolation of mannan and glucan from yeast *Saccharomyces cerevisiae*. Angew. Makromol. Chem..

[20] Byun E.B., Sung N.Y., Kim J.H., Choi J., Matsui T., Byun M.W. (2010). Enhancement of anti-tumor activity of gamma-irradiated silk fibroin via immunomodulatory effects. Chem.-Biol. Interact..

[21] Chen J.R., Hu T.J., Zheng R.L. (2007). Antioxidant activities of Sophora subprosrate polysaccharide in immunosuppressed mice. Int. Immunopharmacol..

[22] Valenzuela A. (1990). The biological significance of determination in the assessment of tissue oxidative stress. Life Sci..

[23] Neilsen F., Mikkelsen B.B., Neilsen J.B., Andersen H.R., Grandjean P. (1997). Plasma malondialdehyde as biomar reference interval and effects of life-style factors. Clin. Chem..

[24] Yagi K. (1987). Lipid peroxides and human diseases. Chem. Phys. Lipids.

[25] Jiau-Jian L., Larry W.O. (1977). Over expression of manganese-containing superoxide dismutase confers resistance to the cyto-toxicity of tumor necrosis factor and/or hyperthermia. Cancer Res..

[26] Ming L., Jill C.P., Jingfang J.N., Edward C., Brash E. (1998). Antioxidant action via p53 mediated apoptosis. Cancer Res..

[27] Yerra R., Malaya G., Upal K.M. (2005). Antitumor activity and *in vivo* antioxidant status of *Mucuna pruriens* (Fabaceae) Seeds against Ehrlich Ascites Carcinoma in Swiss Albino Mice. Iran J. Pharmacol. Ther..

[28] Asakawa T., Matsuhita S. (1980). Colouring conditions of Thiobarbituric acid test for detecting lipid hydroperoxides. Lipids.

[29] Navarini L., Gilli R., Gombac V., Abatangelo A., Bosco M., Toffanin R. (1999). Polysaccharides from hot water extracts of roasted Coffea arabica beans: Isolation and characterization. Carbohydr. Polym..

[30] Luo A.X., Ge Z.F., Fan Y.J., Luo A.S., Chun Z., He X.J. (2011). *In vitro* and *in vivo* antioxidant activity of a water-soluble polysaccharide from *Dendrobium denneanum*. Molecules.

[31] Bradford M.M. (1976). A rapid and sensitive method for the quantitation of microgram quantities of protein utilizing the principle of protein-dye binding. Anal. Biochem..

[32] Yamamoto Y., Nunome T., Yamauchi R., Kato K., Sone Y. (1995). Structure of an exocellular polysaccharide of Lactobacillus helveticus TN-4, a spontaneous mutant strain of Lactobacillus helveticus TY1–2. Carbohydr. Res..

[33] Kumar C.G., Joo H.S., Choi J.W., Koo Y.M., Chang C.S. (2004). Purification and characterization of extracellular polysaccharide from *haloalkalophilic Bacillus* sp. I-450. Enzyme Microb. Technol..

[34] Fan Y.J., He X.J., Zhou S.D., Luo A.X., He T., Chun Z. (2009). Composition analysis and antioxidant activity of polysaccharide from *Dendrobium denneanum*. Int. J. Biol. Macromol..

[35] Pang X.B., Yao W.B., Yang X.B., Xie C., Liu D., Zhang J., Gao X.D. (2007). Purification, characterization and biological activity on hepatocytes of a polysaccharide from *Flammulina velutipes mycelium*. Carbohydr. Res..

[36] Furukawa T., Kubota T., Tanino H., Oura S., Yuasa S., Murate H., Morita K., Kozakai K., Yano T., Hoffman R.M. (2000). Chemosensitivity of breast cancer lymph node metastasis compared to the primary tumor from individual patients tested in the histoculture drug response assay. Anticancer Res..

